# FASN promotes lymph node metastasis in cervical cancer via cholesterol reprogramming and lymphangiogenesis

**DOI:** 10.1038/s41419-022-04926-2

**Published:** 2022-05-21

**Authors:** Qiqiao Du, Pan Liu, Chunyu Zhang, Tianyu Liu, Wei Wang, Chunliang Shang, Jieyu Wu, Yuandong Liao, Yili Chen, Jiaming Huang, Hao Tan, Yunhe Zhao, Meng Xia, Junxiu Liu, Shuzhong Yao

**Affiliations:** 1 Department of Obstetrics and Gynecology, The First Affiliated Hospital of Sun Yat-sen University, Guangzhou, 510080 PR China; 2 Department of Obstetrics and Gynecology, Union Hospital, Tongji Medical College, Huazhong University of Science and Technology, Wuhan, 430022 PR China; 3 Department of Obstetrics and Gynecology, Peking University Third Hospital, Beijing, 100191 PR China; 4 Department of Microbiology, Tumor and Cell Biology, Karolinska Institute, Stockholm, 171 77 Sweden

**Keywords:** Cancer metabolism, Cancer microenvironment, Metastasis, Cervical cancer, Prognostic markers

## Abstract

Cervical cancer (CC) patients with lymph node metastasis (LNM) have a poor prognosis. Clarification of the detailed mechanisms underlying LNM may provide potential clinical therapeutic targets for CC patients with LNM. However, the molecular mechanism of LNM in CC is unclear. In the present study, we demonstrated that fatty acid synthase (FASN), one of the key enzymes in lipid metabolism, had upregulated expression in the CC samples and was correlated with LNM. Moreover, multivariate Cox proportional hazards analysis identified FASN as an independent prognostic factor of CC patients. Furthermore, gain-of-function and loss-of-function approaches showed that FASN promoted CC cell migration, invasion, and lymphangiogenesis. Mechanistically, on the one hand, FASN could regulate cholesterol reprogramming and then activate the lipid raft-related c-Src/AKT/FAK signaling pathway, leading to enhanced cell migration and invasion. On the other hand, FASN induced lymphangiogenesis by secreting PDGF-AA/IGFBP3. More importantly, knockdown of FASN with FASN shRNA or the inhibitors C75 and Cerulenin dramatically diminished LNM in vivo, suggesting that FASN plays an essential role in LNM of CC and the clinical application potential of FASN inhibitors. Taken together, our findings uncover a novel molecular mechanism in LNM of CC and identify FASN as a novel prognostic factor and potential therapeutic target for LNM in CC.

## Introduction

Cervical cancer (CC) is one of the leading causes of death in women globally. According to the worldwide cancer statistics in 2018, there were ~570,000 new CC cases, and 310,000 females die of CC every year [1]. Lymphatic metastasis is the most common type of CC metastasis. Lymphatic metastasis has been reported to be closely related to the prognosis of CC patients. The 5-year overall survival rates for CC patients with 0, 1–2, 3–9, and 10 or more metastatic lymph nodes are 90%, 69%, 57%, and 35%, respectively [2]. Lymph node metastasis (LNM) does not affect the International Federation of Gynecology and Obstetrics (FIGO) staging of CC according to the 2009 FIGO staging principles. However, the FIGO staging principle released in 2018 [3] noted that CC patients with lymph node metastasis should be diagnosed with stage IIIC disease or more, indicating that lymph node metastasis in CC patients has attracted increased attention. Unfortunately, little is known about LNM in CC, and it remains one of the greatest challenges in the field [4].

Lipid metabolism has been revealed to be an essential part of cancer metastasis. Lipids consist of fat (triglyceride, TG) and lipoids (cholesterol, cholesterol ester, and phospholipid). Cancer cells take advantage of the activation of lipid metabolism and corresponding signaling pathways to accelerate malignant progression [5]. Previously, our research revealed that fatty acid metabolism reprogramming contributed to CC lymph node metastasis [6, 7]. To further clarify the relationship between lipid metabolism and CC lymph node metastasis and discover the potential players in this malignant progression, we identified a new potential target, fatty acid synthase (FASN), by applying TCGA dataset analysis and explored its role in LNM of CC in this study.

FASN is a multi-enzyme protein that serves as the key regulator in lipid metabolism, especially fatty acid synthesis. Upregulated FASN expression has been reported to be associated with cancer progression in multiple cancer types, such as prostate cancer, ovarian cancer, breast cancer, and liver cancer [8]. FASN may serve as an oncogenic factor due to its role in regulating cancer cell fatty acid synthesis or driving aberrant lipogenesis in cancer cells [9]. In cervical cancer, studies have utilized gene expression analysis to identify FASN as a potential prognostic or therapeutic target [10, 11]. Nevertheless, the role of FASN in LNM of CC is under investigation.

In the present work, we identified FASN as a key factor contributing to LNM in CC. We showed that FASN is aberrantly upregulated in CC tissue samples and cell lines. High expression of FASN could predict poor prognosis in CC patients and was correlated with lymph node metastasis. Moreover, loss-of-function and gain-of-function experiments demonstrated the key function of FASN in cervical cancer lymph node metastasis. Mechanistically, on the one hand, FASN reprograms cholesterol metabolism and lipid rafts and then activates the c-Src/PI3K/AKT/FAK signaling pathway, contributing to the improved migration and invasion of CC cells. On the other hand, FASN prompts CC cells to secrete IGFBP3 and PDGF-AA to induce lymphangiogenesis. Moreover, the FASN inhibitors C75 and Cerulenin could attenuate CC nodal metastasis in vivo and in vitro. Taken together, our data identify a novel key player in LNM of CC. Genetic targeted therapy or inhibitors based on FASN or this regulatory pathway may shed light on therapeutic options for cervical cancer patients with lymph node metastasis.

## Results

### FASN expression is aberrantly upregulated in cervical cancer cell lines and patient samples

**Fig. 1:**
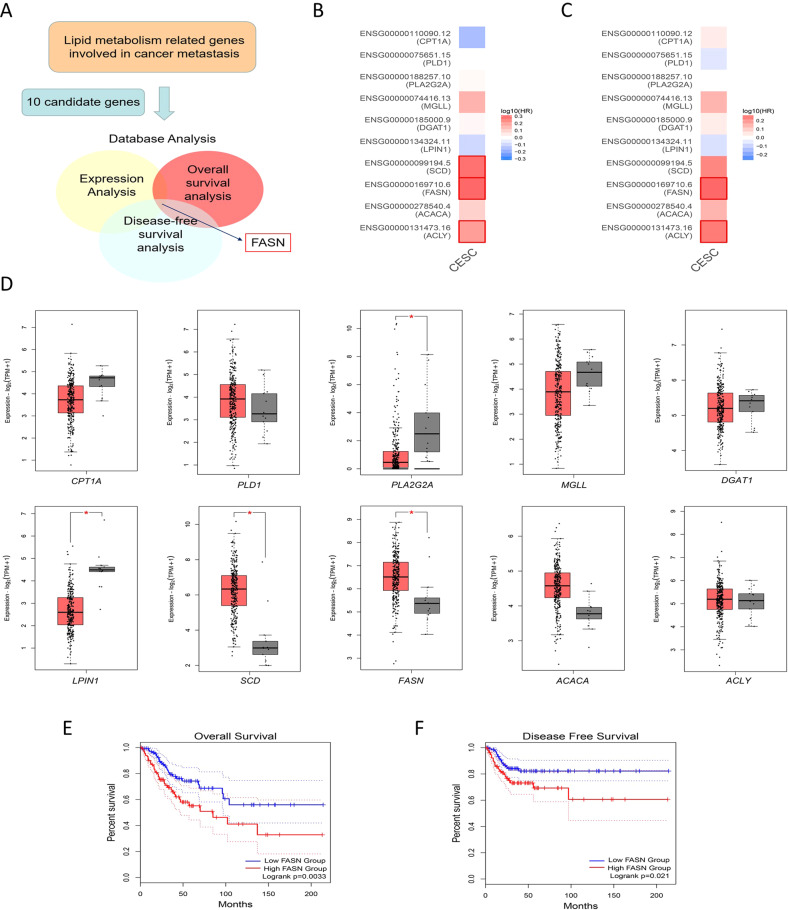
Identification of FASN in CC based on TCGA dataset analysis. **A** Detailed screening workflow using TCGA database to identify FASN. **B** The overall survival map of 10 lipid metabolism-related genes involved in cancer metastasis. The red border indicates the genes with significant *P* values. **C** The disease-free survival map of the same genes in **B**. The red border indicates the genes with significant *P* values. **D** Expression analysis in CC and normal cervical samples in TCGA and GTEx datasets. The red box represents the CC group (*n* = 306), while the gray box shows the normal tissue samples (*n* = 13). **E** Overall survival analysis based on FASN expression level in TCGA database. **F** Disease-free survival analysis based on FASN expression level in TCGA database. *P < 0.05; **P < 0.01; ***P < 0.001.

First, we chose ten genes that encode key enzymes in lipid anabolic and catabolic metabolism associated with metastatic cancer [5, 12, 13]: *ACLY, ACACA*, *FASN*, *SCD*, *LPIN1*, *DGAT1*, *MGLL*, *PLA2G2A*, *PLD1*, and *CPT1A*. Then, we applied the analysis for expression, overall survival (OS), and disease-free survival (DFS) via the online web server GEPIA2 (Fig. 1A). For overall survival analysis, higher expression of *ACLY*, *FASN*, and *SCD* in CC patients was linked to poorer prognosis (*P* values = 0.043, 0.0033, and 0.0076 for *ACLY*, *FASN*, and *SCD*, respectively, Fig. 1B, Fig. S1A). Moreover, patients with higher *ACLY* or *FASN* expression had shorter disease-free survival times (*P* values = 0.048 and 0.021 for *ACLY* and *FASN*, respectively, Fig. 1C, Fig. S1B). *FASN*, *SCD*, *LPIN1*, and *PLA2G2A* were differentially expressed in CC samples compared to normal cervical samples (*P* value = 0.0002, 8.27 × 10^−12^, 2.25 × 10^−11^, and 1.33 × 10^−4^, for *FASN*, *SCD*, *LPIN1,* and *PLA2G2A*, Fig. 1D). Based on these 3 analyses, *FASN* was the only candidate gene that showed meaningful results (Fig. 1B–D). In all, FASN expression was aberrantly upregulated in CC samples (Fig. 1D) and patients with high FASN expression had shorter OS and DFS times (Fig. 1E, F, *P* value = 0.0033, 0.021 respectively for OS and DFS analysis). Therefore, we chose FASN as the focus for further research.

**Fig. 2:**
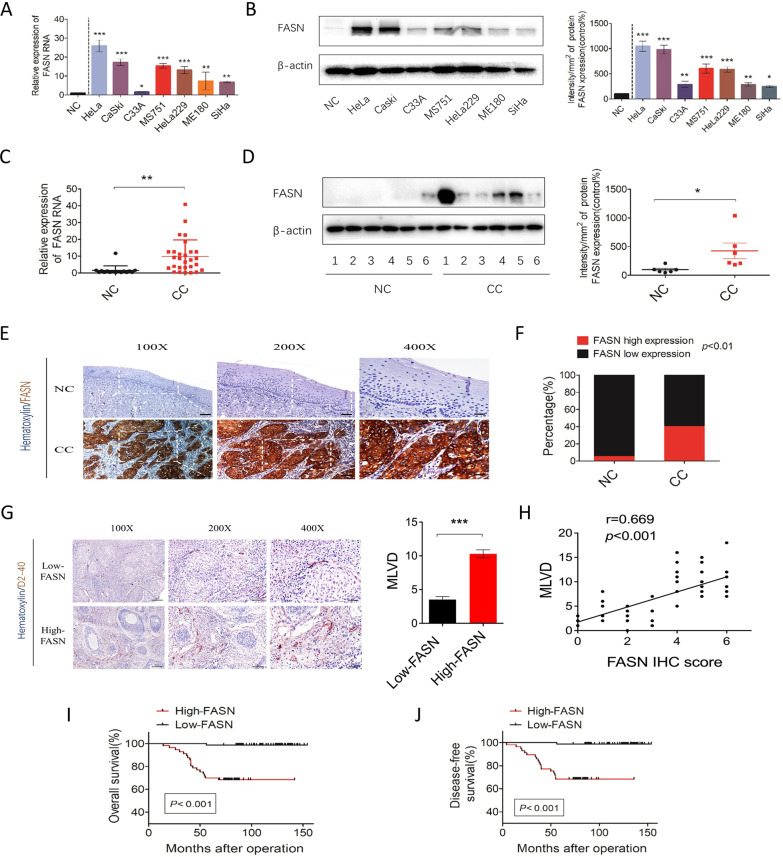
High expression of FASN predicts poor prognosis and is related to MLVD in cervical cancer patients. **A** qRT-PCR analysis of *FASN* mRNA expression levels in various CC cell lines and normal cervical controls. NC = normal cervix. **B** Western blot analysis and quantification of FASN protein levels in CC cell lines and the NC group. **C** qRT-PCR analysis of *FASN* mRNA expression levels in the NC (*n* = 16) and CC (*n* = 28) groups. **D** Western blot analysis and quantification of NC (*n* = 6) and CC (*n* = 6) tissue samples. **E** Representative IHC images of FASN staining in the NC and CC tissue samples. Bar in 100×, 200×, 400 magnifications = 200 µm, 100 µm, 50 µm respectively. **F** Percentage of low and high FASN expression in the NC and CC groups based on the IHC staining results. **G** Representative IHC images of D2-40 staining in the CC groups with low and high FASN expression. Quantification of microlymphatic vessel density (MLVD) is shown. Bar in 100×, 200×, 400 magnifications = 200 µm, 100 µm, 50 µm respectively. **H** Correlation analysis of FASN IHC scores and MLVD. **I** and **J** Overall survival and disease-free survival analysis of CC specimens based on FASN expression. *P < 0.05; **P < 0.01; ***P < 0.001.

Then, we further explored the expression level of FASN in cervical cancer cell lines and patient samples. Compared to the normal cervical samples, all cervical cancer lines, including HeLa, CaSki, C33A, MS751, HeLa229, ME180, and SiHa, showed higher expression of FASN at the mRNA and protein levels (Fig. 2A, B). Similarly, patient samples of cervical cancer (*n* = 28) and normal cervix (*n* = 16) were applied, the results of which indicated that FASN was highly expressed in CC (Fig. 2C, D). To further confirm the high expression level in CC, we also applied immunohistochemistry (IHC) to detect FASN protein levels in patient samples. In 142 patients, 57 CC samples, 40.1% of the total, presented high FASN expression, while only 1 NC sample and 5% of 20 total samples showed high FASN expression (Fig. 2E, F).

Thus, we showed that FASN, one of the key enzymes involved in cancer metastasis, had upregulated expression in CC cell lines and patient samples, indicating that FASN could be an essential oncogenic factor in CC pathology.

### FASN is correlated with lymph node metastasis and predicts poor prognosis in CC patients

Based on the IHC scores, we divided the samples into FASN-low-expression and FASN-high-expression groups and then utilized correlation analysis between FASN expression and the clinicopathological characteristics of CC patients (Table S1). Stromal invasion, pelvic lymph node metastasis, recurrence and vital status at follow-up (*P* values = 0.038, 0.023, <0.001, <0.001, respectively) were found to be closely related to FASN expression. However, other characteristics, such as age, FIGO stage, tumor size, pathologic types, lymph vascular space invasion, differentiation grade, and vaginal involvement, were not relevant. Since FASN expression is related to LNM, we next randomly chose 50 CC samples to determine the microlymphatic vessel density (MLVD). Interestingly, MLVD was increased in the high-FASN-expression group (Fig. 2G). Correlation analysis was applied between MLVD and the FASN IHC scores, the correlation coefficient of which was 0.669, indicating a strong correlation between these factors (Fig. 2H). Moreover, in multivariate Cox proportional hazard model analysis, LNM, recurrence, and FASN were found to be independent prognostic factors (Table S2). And overall survival and disease-free survival analyses were performed to further explore the role of FASN in CC prognosis. The results demonstrated that patients with high expression of FASN had shorter OS and DFS times (*P* value < 0.001, Fig. 2I, J).

### FASN promotes CC cell migration and invasion, lymphangiogenesis in vitro, and lymph node metastasis in vivo

**Fig. 3:**
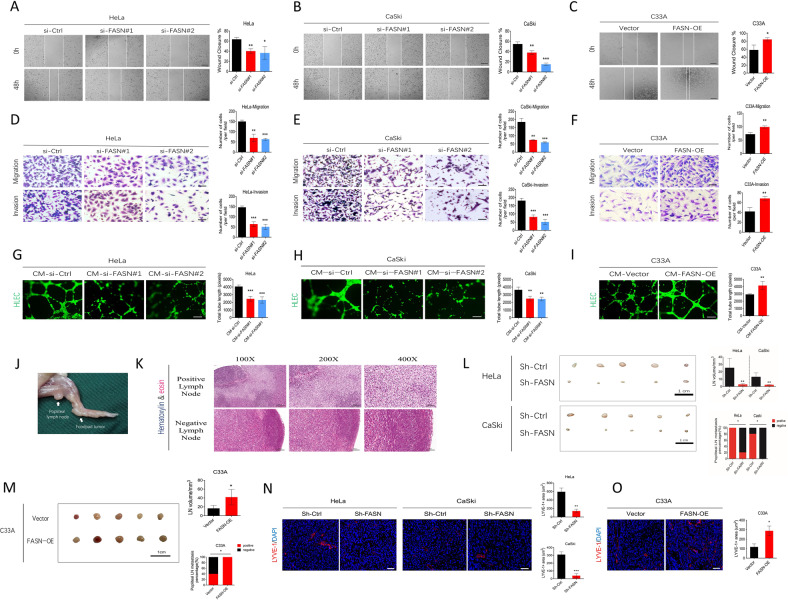
FASN promotes cervical cancer cell migration, invasion, lymphangiogenesis in vitro, and lymph node metastasis in vivo. **A**, **B** The wound healing assay results and quantification in the si-FASN (siRNA#1 and #2) group and si-Ctrl (scramble siRNA) group of HeLa and CaSki cells. Bar = 200 µm. **C** The wound healing assay results and quantification in the Vector (empty vector plasmid) group and FASN-OE (FASN overexpression plasmid) group of C33A cells. Bar=200 µm. **D–F** Transwell migration and invasion results and corresponding quantification in the same group settings as A-C in HeLa, CaSki, and C33A cells. Bar=50 µm. **G**, **H** Assessment of lymphangiogenesis by tube formation assays in the CM-si-FASN (FASN siRNA#1 or #2-treated tumor culture medium) and CM-si-Ctrl (control scrambled siRNA-treated tumor culture medium) groups of HeLa and CaSki cells. Total tube length was quantified. Bar=100 µm. **I** Tube formation assays performed in the CM-Vector and CM-FASN-OE groups of C33A cells. **J**. In vivo mouse model of CC lymph node metastasis. Arrow indicates the footpad tumor and popliteal lymph node. **K** Representative H&E staining images of positive and negative metastatic lymph nodes. Bar in 100×, 200×, 400 magnifications = 200 µm, 100 µm, 50 µm respectively. **L** Gross popliteal lymph node specimen and quantification of popliteal LN volume and metastasis percentage is indicated in the HeLa- or CaSki-sh-Ctrl and HeLa- or CaSki-sh-FASN groups. Bar=1 cm. **M** Gross popliteal lymph node specimen and quantification of popliteal LN volume and metastasis percentage are indicated in the C33A-vector and C33A-FASN-OE groups. Bar=1 cm. **N** LYVE-1 immunofluorescence staining showed the lymphatic vascular density in tumors of the sh-Ctrl and sh-FASN groups. Bar = 25 µm. **O** LYVE-1^+^ lymphatic vascular density in tumors of C33A-vector and C33A-FASN-OE groups. *P < 0.05; **P < 0.01; ***P < 0.001.

Expression analysis, correlation analysis, and OS and DFS analysis identified the key role of FASN in LNM of CC. Next, we mainly focused on the effect of FASN on the migration and invasion of CC cells and lymphangiogenesis in vitro. Based on the FASN expression level of CC cell lines, HeLa and CaSki cells showed the highest expression among these cell lines, so they were chosen for subsequent loss-of-function experiments. Knockdown of FASN via siRNA was applied in these two cell lines. Wound healing, Transwell migration and invasion assays showed that fewer migrating HeLa cells were observed in the si-FASN group (knockdown group) than in the si-Ctrl group (scrambled siRNA group) (Fig. 3A, D). Similar results were observed in CaSki cells (Fig. 3B, E). To confirm the effect of FASN on CC cell migration and invasion, we performed gain-of-function experiments with C33A cells, which expressed comparatively low FASN among those cell lines. After treatment with the FASN overexpression plasmid, C33A cells presented increased migration and invasion compared to that of the control group (Fig. 3C, F). Moreover, we aimed to verify that the regulation of FASN in cervical cancer cells could affect the interaction between tumor cells and human lymphatic endothelial cells (HLECs). We observed that tube formation of HLECs was impaired in the FASN knockdown group (Fig. 3G, H), while higher expression of FASN (FASN-OE group) benefited HLEC tube formation (Fig. 3I), proving that FASN could contribute to lymphangiogenesis.

Furthermore, we evaluated the role of FASN in LNM of CC in vivo. First, we established the LNM model by injecting CC cells into the foot pad (Fig. 3J), and the metastatic status was confirmed by HE staining (Fig. 3K). Interestingly, we observed shrinkage of lymph node volume in the sh-FASN group (FASN knockdown group) compared to the corresponding control group (Fig. 3L). Moreover, the popliteal lymph node metastasis percentage was significantly decreased in the sh-FASN group in both the HeLa and CaSki cells (Fig. 3L). LYVE-1 staining showed less lymphatic vascular structure in the tumor site in the sh-FASN group (Fig. 3N), providing evidence that FASN contributes to LNM in CC. On contrast, FASN overexpression could lead to increased popliteal lymph node metastasis percentage, lymph node volume, and lymphatic vascular structure density (Fig. 3M, O).

### FASN regulates cholesterol reprogramming

**Fig. 4:**
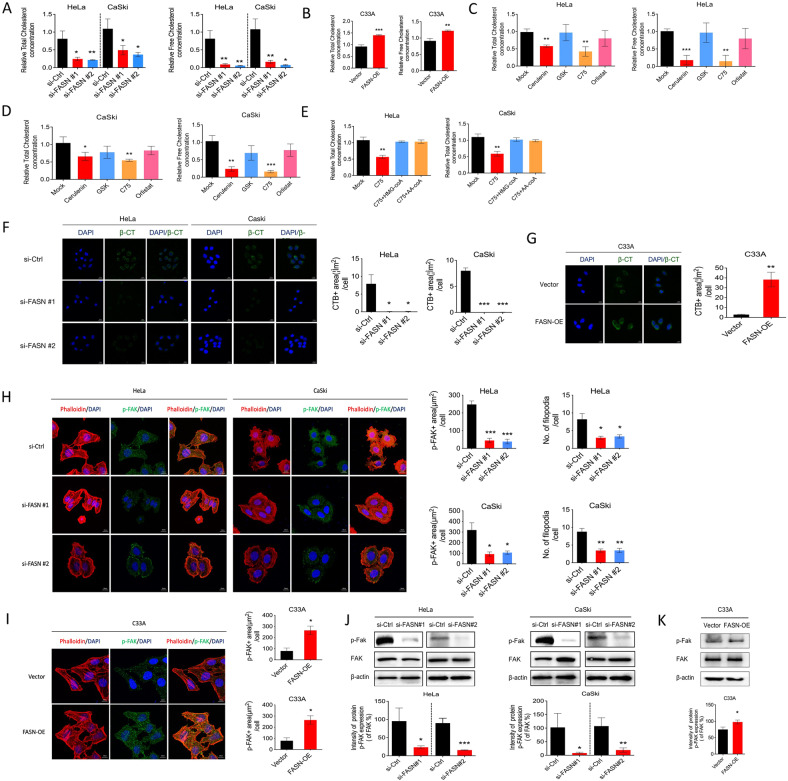
FASN regulates cholesterol reprogramming, consequently leading to lipid rafts and actin skeleton remodeling. **A** Assessment of total and free cholesterol concentration in si-FASN (FASN siRNA#1 and #2) and si-Ctrl groups of the HeLa and CaSki cells. **B** Total and free cholesterol concentration in C33A-vector and C33A-FASN-OE groups. **C**, **D** Assessment of total and free cholesterol concentration in the HeLa and CaSki cells treated with different FASN inhibitors (Cerulenin, GSK, C75 and Orlistat). **E** Total cholesterol concentration in the HeLa and CaSki cells treated with Mock, C75, C75 + HMG-coA, C75 + AA-coA. **F**, **G** Lipid raft staining images and corresponding positive areas were analyzed in the same settings in **A**, **B**. **H** F-actin and phospho-FAK IF staining is shown, and p-FAK positivity and the number of filopodia were quantified in si-FASN (FASN siRNA #1 and #2) and si-Ctrl groups of HeLa and CaSki cells. **I** F-actin and phospho-FAK IF staining, quantification of p-FAK^+^ area and filopodia number were indicated in C33A-vector and C33A-FASN-OE groups. **J**, **K** Western immunoblot analysis of p-FAK, FAK, and β-actin after FASN knockdown (siRNA#1 and #2) and FASN overexpression (FASN-OE). *P < 0.05; **P < 0.01; ***P < 0.001.

The link between FASN and fatty acid metabolism has been widely acknowledged. However, whether FASN could lead to other metabolic processes remains largely unknown. Recent research revealed that cholesterol was highly involved in cancer metastasis [14], and FASN was found to be linked with cholesterol metabolism [15]. Therefore, we intended to explore whether FASN could affect cholesterol metabolism. We tested the total amount of cholesterol and free cholesterol in HeLa and CaSki cells and found that it was decreased after FASN inhibition by FASN-targeted siRNA while FASN overexpression led to increased total and free cholesterol (Fig. 4A, B). Furthermore, 4 FASN inhibitors, which target different domains of FASN, were applied to further prove the effect of FASN on cholesterol metabolism, the results of which demonstrated a similar pattern to that of C75 and Cerulenin treatment but not GSK2194069 and orlistat (Fig. 4C, D). C75 and Cerulenin could target the ketoacyl synthase (KS) domain while the GSK targets ketoacyl reductase (KR) domain and Orlistat targets the thiosterase (TE) domain, which indicated that C75 and Cerulenin could inhibit acetoacetyl-CoA while GSK and Orlistat could not inhibit acetoacetyl-CoA. Besides, the addition of acetoacetyl-CoA or a metabolite of cholesterol synthesis, HMG-CoA could rescue the inhibition of cholesterol by C75 (Fig. 4E), which supports that acetoacetyl-CoA or HMG-CoA are the key intermediate metabolites here. The results we presented demonstrated that enzymatic domains of FASN that were at the KS but not KR or TE were essential for inhibiting cholesterol reprogramming in cervical cancer cells.

Cholesterol reprogramming could affect cell migration by actin remodeling and lipid rafts [16–18]. Thus, these two important reprogramming processes were assessed. After FASN knockdown, fewer lipid rafts were observed in both HeLa and CaSki cells (Fig. 4F). In the actin remodeling experiment, the results showed that phosphor-FAK (p-FAK) colocalized in the filopodia of CC cells (Fig. 4H). Furthermore, the p-FAK-positive area and the number of filopodia were both decreased after FASN knockdown with siRNA (Fig. 4H), indicating that FASN regulated actin remodeling. Western blot results also indicated the impairment of p-FAK expression in the si-FASN group of HeLa and CaSki cells (Fig. 4J). While the gain of function experiments proves that FASN overexpression increased lipid rafts area, p-FAK-positive area, the number of filopodia, and p-FAK expression (Fig. 4G, I, K).

### FASN regulates the c-Src/PI3K/AKT/FAK signaling pathway cascade via lipid rafts

**Fig. 5:**
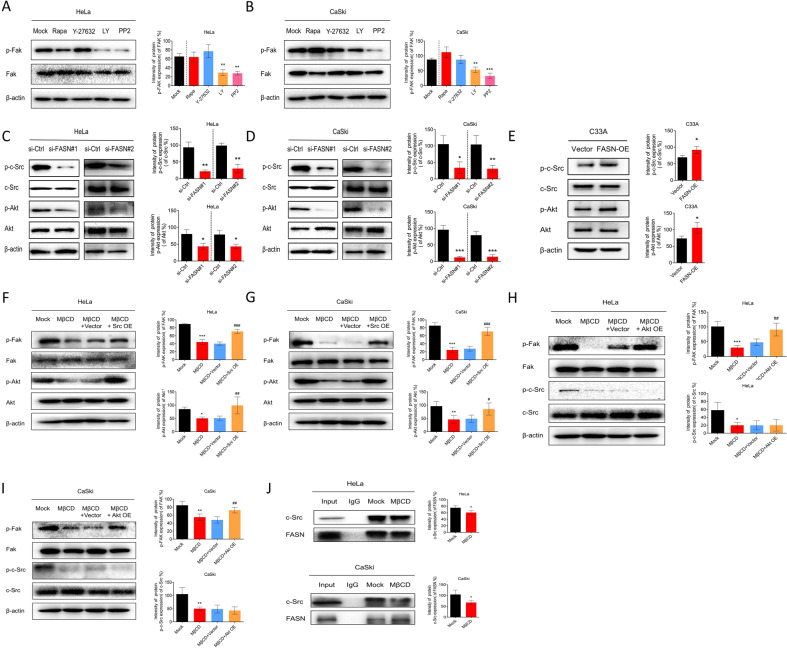
FASN regulates the c-Src/PI3K/AKT/FAK cascade via lipid rafts. **A**, **B** Signaling pathway screening by various pathway inhibitors (Rapamycin-mTOR pathway inhibitor 50 nM, Y-27632-ROCK pathway inhibitor 30 µM, LY294002-PI3K/AKT pathway inhibitor 50 µM, PP2-c-SRC pathway inhibitor 10 mM) in HeLa and CaSki cells. **C**, **D** Western blot analysis of p-c-Src, c-Src, p-Akt, and Akt expression in the si-FASN (#1 and #2) and si-Ctrl groups. **E** Western blot analysis of p-c-Src, c-Src, p-Akt, and Akt expression in the C33A-FASN-OE and C33A-vector groups. **F**, **G** Rescue experiments by applying a c-Src overexpression plasmid (Src OE) and MβCD. p-Fak, Fak, p-Akt and Akt expression levels were detected and analyzed. **H**, **I** Rescue experiments by applying Akt overexpression plasmid (Akt OE) and MβCD. p-Fak, Fak, p-Akt, and Akt expression levels were detected and quantified. **J** Co-IP experiments were conducted to analyze the interaction between FASN and c-Src with or without a lipid raft inhibitor. Protein extracts were immunoprecipitated with an anti-FASN antibody and the immunoprecipitations were assayed for c-Src. *P < 0.05; **P < 0.01; ***P < 0.001; ^#^*P* < 0.05; ^##^*P* < 0.01; ^###^*P* < 0.001, MβCD + Src/Akt OE vs. MβCD + Vector.

Lipid rafts have been reported to be essential platforms for signaling regulation [19]. Thus, for a detailed mechanistic study, we first chose to clarify whether various signaling pathways, mTOR, ROCK, PI3K/AKT, and c-Src, which have been found to be related to cervical cancer tumorigenesis or progression [20–22], were involved. We intended to explore the upstream signaling pathway in regulating p-FAK. Inhibitors of mTOR, ROCK, PI3K/AKT, and c-Src signaling pathways were used to screen the signaling pathways. We found that p-FAK expression remained unchanged when mTOR and ROCK inhibitors were applied, while c-Src and PI3K/AKT inhibitors significantly decreased its expression level (Fig. 5A, B). Next, we focused on the c-Src and PI3K/Akt pathways. When FASN was silenced, p-FAK, p-c-Src (phosphor-c-Src), and p-Akt (phosphor-Akt) expression was sequentially downregulated (Fig. 5C, D) while their expressions were upregulated by FASN overexpression (Fig. 5E). Moreover, lipid raft inhibition by MβCD suppressed p-FAK, p-c-Src and p-Akt expression, indicating that lipid rafts may provide a platform for c-Src and Akt signaling pathway activation (Fig. 5F, G).

To further discuss the relationship between these signaling pathways, we utilized rescue experiments. When we disturbed the lipid rafts with MβCD and overexpressed c-Src with the plasmid, the upregulation of p-FAK and p-Akt expression was observed (Fig. 5F, G). However, when the MβCD and Akt overexpression plasmids were applied at the same time, we could only detect the upregulation of p-FAK expression but not higher p-c-Src expression levels, indicating that c-Src was upstream of the Akt pathway (Fig. 5H, I). Therefore, c-Src could be this signaling pathway cascade trigger. In this sense, we also intended to clarify whether FASN could interact with c-Src and whether lipid rafts could serve as the interaction platform. Co-IP results demonstrated that FASN interacted with c-Src in a quiescent state in both HeLa and CaSki cells. When MβCD was utilized, the interaction between FASN and c-Src was impaired (Fig. 5J). Taken together, the data showed that FASN could regulate lipid rafts and then activate the c-Src/PI3K/Akt/FAK signaling pathway cascade.

### FASN upregulates PDGF-AA/IGFBP-3 secretion in CC cells to accelerate lymphangiogenesis

**Fig. 6:**
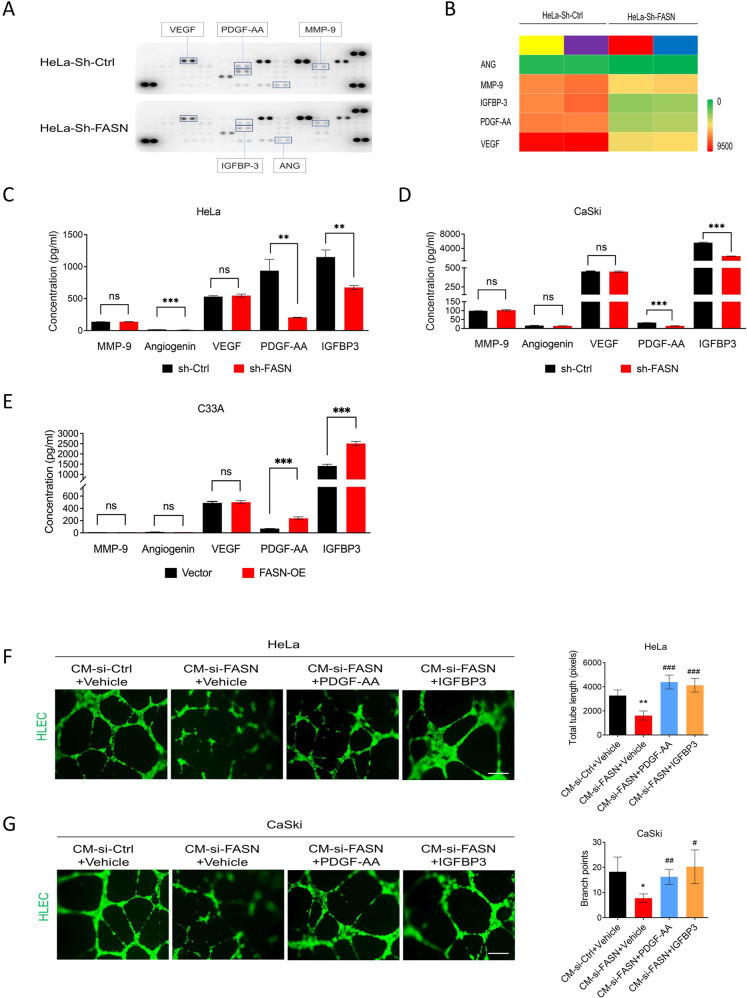
FASN induces lymphangiogenesis by secreting PDGF-AA and IGFBP-3. **A** Human angiogenesis array kit results showed five differentiated angiogenesis-related factors in the HeLa-sh-Ctrl and HeLa-sh-FASN groups. Each group contained three replicates of the culture supernatant mixture. **B** Heatmap of these five factors in A based on pixel analysis. **C**, **D** ELISA validation of MMP-9, Angiogenin, VEGF, PDGF-AA, and IGFBP-3 in the culture supernatant of the FASN-shRNA-treated HeLa and CaSki cells. **E** ELISA validation of MMP-9, Angiogenin, VEGF, PDGF-AA, and IGFBP-3 in the culture supernatant of the C33A-FASN-OE and C33A-vector groups. **F**, **G** Rescue experiments showed that the addition of PDGF-AA and IGFBP-3 could rescue the impaired effect of FASN knockdown on lymphangiogenesis. Tube length in tube formation assays was quantified. *P < 0.05; **P < 0.01; ***P < 0.001; ^#^*P* < 0.05; ^##^*P* < 0.01; ^###^*P* < 0.001, CM-si-FASN-PDGF-AA/IGFBP-3 vs. CM-si-FASN-vehicle.

One important process in lymphatic metastasis is local lymphangiogenesis in cancer. Researchers have discovered that the crosstalk between tumor cells and lymphatic endothelial cells is an essential part of cancer metastasis and that growth factors could be messengers between them [23]. Thus, in this part, we mainly explored which growth factors could be regulated by FASN to promote lymphangiogenesis. First, the screening of potential growth factors was performed by applying a Human Angiogenesis Array Kit. We chose the angiogenesis array kit for the following reasons: (1). Some families of cell surface receptors, such as integrins α4β1 and α2β1 [24], have been reported to be regulators of both tumor angiogenesis and lymphangiogenesis, indicating that angiogenesis-related proteins may also have similar effects on lymphangiogenesis. (2) Some angiogenesis-related growth factors included in this array kit, including Ang-1, Ang2, PDGF-AA/BB [25], VEGF-C, and VEGF-A, were shown to play a role in lymphangiogenesis [26]. Based on the expression of angiogenesis-related factors, we found the five top differentiated factors: angiogenin (ANG), VEGF, MMP-9, PDGF-AA, and IGFBP-3 (Fig. 6A, B, Fig. S2). ELISA validation was then utilized to narrow down the growth factors. Only PDGF-AA and IGFBP-3 levels were confirmed to be truly downregulated after FASN silencing in both HeLa and CaSki cells (Fig. 6C, D). Besides, more PDGF-AA and IGFBP-3 were detected in FASN-OE group than in the corresponding control (Fig. 6E). Furthermore, we performed rescue experiments to confirm the effect of PDGF-AA and IGFBP-3 on lymphangiogenesis. After FASN knockdown, the total length was significantly decreased, while PDGF-AA and IGFBP-3 rescued this impairment (Fig. 6F, G).

### The FASN inhibitors C75 and Cerulenin suppress nodal metastasis in vivo and in vitro

**Fig. 7:**
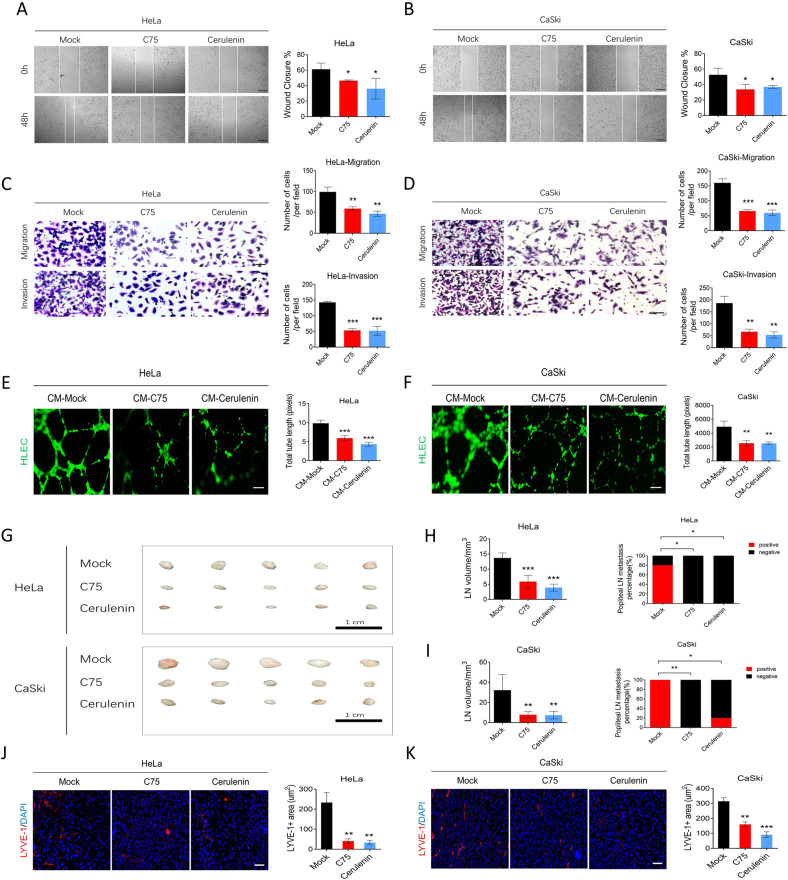
FASN inhibitors suppress cervical cancer cell migration, invasion, lymphangiogenesis in vitro, and lymph node metastasis in vivo. **A**, **B** The wound healing assay results in the mock-, C75-, and Cerulenin-treated HeLa and CaSki cells. Bar = 200 µm. **C**, **D** Transwell migration and invasion results in the same settings as **A**, **B**. Bar=50 µm. **E**, **F** Assessment of lymphangiogenesis by tube formation assays in the CM-mock (mock vehicle-treated tumor culture medium) and CM-C75 or CM-Cerulenin (C75- or Cerulenin-treated tumor culture medium) groups and the total tube length. Bar = 100 µm. **G** A gross popliteal lymph node specimen is shown. Bar=1 cm. **H**, **I** LN volume and metastasis percentage were quantified in the mock, C75, and Cerulenin groups using both cell lines injected into the CC lymph node mouse model. **J**, **K** LYVE-1 immunofluorescence staining showed the lymphatic vascular density in the same setting groups as **A**, **B**. Bar=25 µm. *P < 0.05; **P < 0.01; ***P < 0.001.

Since we proved that FASN could promote LNM in CC, we further explored its application potential in CC patients in the future. Based on the inhibition results in Fig. 4B, E, we chose C75 and Cerulenin to examine the effect of existing FASN inhibitors on LNM in CC. An in vitro wound healing assay revealed that C75 and Cerulenin could impair the migration of HeLa and CaSki cells (Fig. 7A, B). Transwell migration and invasion experiments further confirmed the effect of C75 and Cerulenin on migration and invasion (Fig. 7C, D). Moreover, lymphangiogenesis was impaired when C75 and Cerulenin were applied (Fig. 7E, F). In vivo, C75 and Cerulenin remarkably suppressed the lymph node volume and popliteal lymph node metastasis percentage (Fig. 7G–I). Moreover, lymphatic vascular density was reduced in the C75 and Cerulenin groups compared to the control group (Fig. 7J, K). The data indicated that C75 and Cerulenin could effectively inhibit LNM in CC .

**Fig. 8:**
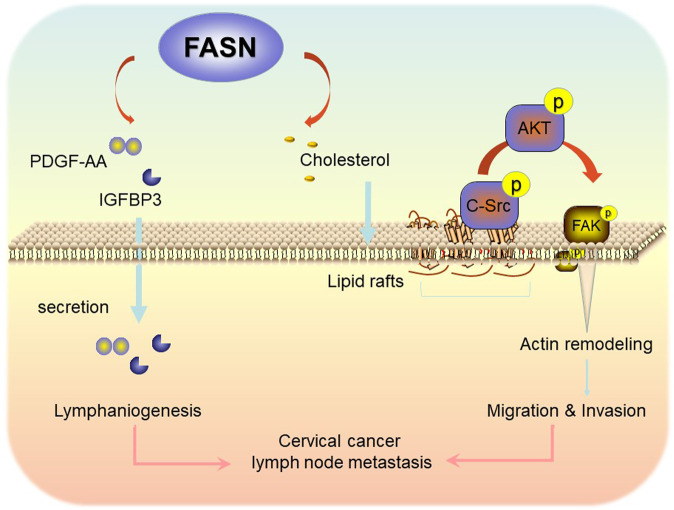
FASN promotes lymph node metastasis in cervical cancer via cholesterol reprogramming and lymphangiogenesis. Proposed schematic representation of the mechanism demonstrating that FASN promotes lymph node metastasis in cervical cancer via cholesterol reprogramming and lymphangiogenesis.

## Discussion

Lymph node metastasis usually predicts poor prognosis in CC patients, and there are limited therapeutic options, which makes it a serious obstacle for these patients. Hence, a detailed demonstration of how lymph node metastasis occurs and the driving factors behind this process are urgently needed. Regrettably, little is known about LNM in CC. Some interesting studies have shown that lipid metabolism may be instrumental in cancer metastasis. Our laboratory has demonstrated that fatty acid metabolism reprogramming is conducive to CC lymph node metastasis [6, 7]. Independent studies from another laboratory clarified that dietary oleic acid promoted CC metastasis [27], supporting the fact that lipid metabolism plays a role in CC metastasis. Therefore, as a starting point, we examined lipid metabolism-related genes to explore their expression level and prognostic value in CC using TCGA dataset analysis. Finally, we identified FASN as our focus in this study.

In the present study, we showed that FASN could promote CC lymph node metastasis. Some independent studies have shown that FASN serves as an oncogenic factor and enhances LNM in multiple types of cancer [28–30]. Then, we further explored the detailed molecular mechanism. How does lymphatic metastasis occur? This question is under investigation. One convincing possibility is that the improved migration and invasion of cancer cells allows them to more easily enter the lymphatic system or blood vascular system [31]. Another possibility is that the interaction and communication between cancer cells and HLECs lead to increased local lymphangiogenesis [32]. As a result, we explored the detailed mechanism, focusing on the driving force of the increased migration and invasion of cancer cells and the interaction between cancer cells and HLECs.

We proved that FASN modulated cholesterol metabolism, leading to lipid raft reprogramming and actin remodeling and contributing to enhanced migration and invasion of CC cancer cells. As a key enzyme in lipid metabolism, FASN regulates the anabolic and catabolic processes of many lipids, including cholesterol, which is the main component of lipid rafts. Here, we provided data showing that inhibition of FASN suppressed cellular total and free cholesterol. This interesting mechanism is similar to one in a recent study showing that FASN regulated cholesterol metabolism in hepatocarcinoma [33]. Another study likewise presented the unexpected link between FASN and cholesterol metabolism [15]. Our findings and these studies identify a novel mechanism of FASN in cancer lipid metabolism, providing more possibilities for future investigation. Lipid rafts, which consist of cholesterol and phospholipids, are known as the lipid microdomains that are present in the external layer of the plasma membrane. Lipid rafts are regarded as the signaling hub in cancer cell invasion [34]. Similarly, our data showed that in CC cancer cells, lipid raft disturbance led to c-Src/PI3K/AKT/FAK signaling pathway disorder. Actin remodeling is another metastasis-related morphological change we evaluated here. Actin remodeling is usually an essential step for cancer cell migration, marked by an increase in filopodia and lamellipodia [35]. The importance of FAK-mediated actin remodeling in cancer migration has been revealed by our previous study [36] and some recent studies [37, 38].

Lymphangiogenesis based on the communication between cancer cells and HLECs is another focus of this study. Many studies have shown that growth factors, such as VEGF-C and VEGF-D, secreted by cancer cells could affect HLEC function to induce lymphangiogenesis [39]. In cervical cancer, VEGF-C and galecin-1 were shown to be lymphangiogenesis-related factors [40, 41]. Nevertheless, the tumor-secreted factors that stimulate HLECs to induce tumor lymphangiogenesis in CC lymph node metastasis are unclear. Here, we screened several factors and found that FASN could induce CC cells to secrete PDGF-AA and IGFBP-3 to affect lymphangiogenesis. PDGF-AA was shown to induce lymphangiogenesis, and IGFBP-3 was indicated to be related to lymph node metastasis in oral squamous cell carcinoma and thyroid carcinoma [42, 43]. In this section, we showed that FASN could modulate PDGF-AA and IGFBP-3 secretion to affect lymphangiogenesis, which will hopefully lead to the identification of more messengers in the communication between cancer cells and HLECs.

Another interesting finding is that the FASN inhibitors C75 and Cerulenin could significantly impair lymphatic metastasis in vivo and in vitro, indicating the potential application of FASN inhibitors in CC targeted therapy. Multiple FASN inhibitors, including C75, Cerulenin, and orlistat, have shown antitumor activity in cancer cell lines or mouse models [44, 45]. However, these compounds have not been tested in patients due to limitations such as their side effects or pharmacologic properties [46]. The new generation of molecules, such as TVB-2640 IPI-9119, are in development, and TVB-2640 has been tested clinically. Whether the new generation of FASN inhibitors could be effective in reducing CC lymphatic metastasis needs further exploration.

In summary, our findings demonstrate that FASN, as a prognostic factor of CC, promotes lymph node metastasis via cholesterol reprogramming and lymphangiogenesis. A detailed mechanistic investigation clarified that FASN regulates CC cell migration and invasion via lipid raft-related c-Src/PI3K/AKT/FAK signaling pathway disorder. In addition, FASN promotes PDGF-AA and IGFBP-3 secretion to benefit lymphangiogenesis. Therefore, we not only identified a new therapeutic biomarker for CC patients with lymph node metastasis but also provided a novel regulatory molecular mechanism involving cholesterol reprogramming and PDGF-AA/IGFBP-3 secretion (Fig. 8). Our results may provide mechanistic insights and a potential novel therapeutic strategy for CC patients with lymph node metastasis.

## Materials and methods

### GEPIA dataset

Gene Expression Profiling Interactive Analysis 2 (GEPIA2) is a newly enhanced interactive web server for analyzing RNA sequencing expression data from The Cancer Genome Atlas (TCGA) and the Genotype-Tissue Expression projects [47]. CESC was the focus of this study and includes 306 tumor samples and 13 normal cervical samples. Expression DIY with box plots was implied, with the |log2FC| cutoff set as 1 and the *p* value cutoff set as 0.01. Overall survival and disease-free survival analyses were performed by applying the group cutoff as the median.

### Cell culture

The human cervical cancer cell lines SiHa, CaSki, C33A, MS751, HeLa229, ME180, and HeLa were obtained from Shanghai Institutes for Biological Sciences (Chinese Academy of Sciences, China). Cell media were as follows: DMEM/F-12 (HyClone) medium for HeLa and SiHa cells, RPMI-1640 (Gibco) medium for CaSki cells, MEM (Gibco) medium for C33A, MS751, and HeLa229 cells, and McCoy’s 5a Medium Modified (Sigma) for ME180 cells. Incubation was carried out in a medium containing 10% fetal bovine serum (FBS), 0.2 UI/mL insulin, L-glutamine, and penicillin and streptomycin under a 5% CO_2_ atmosphere at 37 °C.

### RNA isolation and qPCR

A QIAzol-chloroform-based protocol was used for the extraction of RNA from tissue samples, while lysis buffer was used from the isolation kit for cell samples. All kits were purchased from TaKaRa (Dalian, China). All procedures were performed according to the manufacturer’s instructions. Total RNA concentrations were measured with a NanoDrop2000, and an equal amount of RNA from each sample was used for cDNA synthesis using a RevertAid cDNA synthesis kit. The cDNAs were subsequently used for qPCR analysis with Power SYBR Green Master Mix using the Step One Plus Real-Time PCR System. The qPCR primer sequences were as follows: *FASN*-forward 5′-CGCGTGGCCGGCTACTCCTAC-3′ and *FASN*-reverse 5′-CGGCTGCCACACGCTCCTCT-3′.

### Immunoblotting

Cell lysates were separated by SDS-PAGE, followed by wet transfer onto 0.45 µm PVDF membranes, which were then blocked with 5% BSA in PBST solution for 30 min. Specific primary antibody incubation was conducted overnight at 4 °C. The antibodies used were as follows: anti-phospho-Y397-Fak (ab81298, Abcam, USA), anti-FAK (ab131435, Abcam, USA), anti-Tyr416-phosphor-c-Src (#2010s, CST), anti-c-Src (#2108s, CST), anti-Ser473-phospho-Akt (#4060s, CST), anti-Akt (#9272, CST, Danvers, MA), anti-FASN (#14979-1-AP, Proteintech, Rosemont, USA), anti-β-actin (#sc-81178, Santa Cruz, USA). Secondary antibody was incubated for 1 h at RT. Immunodetection was accomplished using enhanced chemiluminescence. The band intensity was determined with ImageJ software. All the original western blot pictures were provided in the “Supplemental Material original western blots”.

### Immunohistochemistry (IHC)

Paraffin-embedded tissue samples of 5-µm sections were subjected to IHC staining. A series of graded alcohols were used for slides deparaffinized in xylene and rehydrated. Blocking with 4% goat serum was performed before incubation with a primary antibody (anti-FASN, #14979-1-AP, Proteintech; anti-D2-40, #67432-1, Proteintech) overnight at 4 °C. HRP-conjugated secondary antibody was incubated for 1 h at room temperature. Then, DAB detection, hematoxylin, dehydration in graded ethanol, and mounting were applied. The IHC scores (Range from 0 to 6) were evaluated as previously described [6, 7]. The results of IHC were scored by adding the staining area (0 = no, 1 = less than 30%, 2 = between 30% and 60%, 3 = between 60% and 100% stained cells) and the staining intensity (0 = no, 1 = weak, 2 = moderate, 3 = strong staining). Two experienced pathologists evaluated scores in a blinded fashion. A staining score of 4 was defined as the cutoff. Thus, patients with different levels of expression were divided into low- and high-staining groups.

### Transfection experiments

HeLa and CaSki cells were transfected with siRNA/shRNA/plasmids using Lipofectamine (Thermo Fisher, USA) or lentivirus according to the protocol. The siRNAs and plasmids used included FASN siRNA (#stB0006298, RiboBio Co., Ltd., China), pcDNA3 vector (empty negative control), pcDNA3-c-Src plasmid (Addgene, Cambridge, MA), Akt plasmid (#CH846646, Vigene Biosciences, China), pLKD-CMV-mCherry-2A-Puro-U6-shRNA-FASN (#HYKY-180509017, Obio Technology, China), and pLKD-CMV-mCherry-2A-Puro-U6-shRNA-Ctrl (Obio Technology, China). Cells (60% confluent) were serum-starved for 6–8 h. Incubation with 80 nM siRNA/plasmids was performed for 12 h in Opti-MEM Media (Thermo Fisher, MO, USA). Full medium (10% FBS) was then added for 36 h before experiments or functional assays.

### Wound healing assays and transwell migration/invasion assays

Cells were seeded in a 12-well plate and then transfected with siRNA or plasmid or pretreated with C75 (50 µM) and Cerulenin (50 µM) inhibitors for one hour until reaching 100% confluence. A scratch presenting a wound was made inside the well, and then, the cells were cultured for another 48 h to observe cell migration. The closure area was quantified to indicate the cell migration. For Transwell migration/invasion assays, a total of 2 × 10^4^ cells in 200 µl of serum-free medium were seeded in the upper chamber of 8-µm Transwell inserts (BD Biosciences, Franklin Lakes, NJ, USA). The lower chamber was filled with 10% FBS medium. Twenty-four hours later, the remaining cells in the upper chamber were removed. The cells adhering to the membrane were fixed with methanol for 15 min and then stained with 0.1% crystal violet (KeyGEN Biotech, Nanjing City, China) for 30 min. For the invasion assays, the upper chamber was precoated with 50 µl of Matrigel (#356234, BD Biosciences), and 2 × 10^5^ cells in 200 µl of serum-free medium were seeded. The rest of the procedure was the same as that for the migration assays.

### Tube formation assay

2 × 10^4^ HLECs (human lymphatic endothelial cells) were seeded into a 96-well plate that had been precoated with 50 µl Matrigel (#356234, BD Biosciences). After treatment with cancer cell culture medium, cells were stained with calcein-AM (10 mM) for 30 min. Tube formation was visualized, and pictures were taken. The total length was quantified via ImageJ with angiogenic analysis.

### In vivo nude mouse lymph node metastasis model

Nude mice were maintained in SPF conditions at the Department of Sun Yat-sen University Animal Center strictly according to the institution’s guidelines. All animal work in this study was approved by the Animal Ethical and Welfare Committee of Sun Yat-sen University. Female BALB/c nude mice (4–6 weeks of age, 18–20 g) were randomly divided into a control group and a treatment group. Cells (1 × 10^7^) stably silenced by FASN-shRNA lentivirus were injected into a food pad. C75 (10 mg/kg, #C5490, Sigma-Aldrich) and Cerulenin (30 mg/kg, #C2389, Sigma-Aldrich) were intraperitoneally injected every other day for 2 weeks. The popliteal lymph nodes were removed for hematoxylin and eosin staining. The lymph node volumes were calculated with the following formula: volume (mm^3^) = (length [mm]) × (width [mm])^2^ × 0.52.

### Lipid raft labeling

This assay was performed according to the instructions (Vybrant^®^ Alexa Fluor® 555 Lipid Raft Labeling Kit, #V-34404, Molecular Probes, Inc.) HeLa and CaSki cells were seeded on coverslips and then labeled with fluorescent CT-B conjugate for 10 min at 4 °C. Then, the CT-B-labeled lipid rafts were crosslinked with anti-CT-B antibody at 4 °C for 15 min. The cells were fixed in 4% PFA for 15 min at 4 °C and then permeabilized with 0.1% Triton X-100 for 10 min. The cells were washed and mounted.

### Cell immunofluorescence

HeLa, CaSki, and C33A cells were seeded on coverslips and exposed to treatments. Cells were fixed with 4% paraformaldehyde for 20 min at room temperature and permeabilized with 0.1% Triton X for 5 min. Blocking was performed with 5% bovine serum albumin (BSA) for 20 min. Cells were incubated with primary antibodies against phospho-Y397-Fak (ab81298, Abcam, USA) overnight at 4 °C and then with FITC-conjugated secondary antibody at RT for 1 h. After washing, F-actin was stained with Acti-stain 555 phalloidin (#PHDH1, Cytoskeleton, Inc.) The nuclei were counterstained with 4′-6-diamidino-2-phenylindole (Sigma-Aldrich). The coverslips were mounted with Vectashield mounting medium (Vector Laboratories, CA). Immunofluorescence was visualized using an Olympus BX41 microscope.

### Cholesterol detection

Measurement of total cholesterol and free cholesterol was performed according to a cholesterol/cholesterol ester detection kit (#ab102515, Abcam). Cells (10^6^) were extracted with 200 μl of CHCl3:IPA:NP-40 (7:11:0.1) in a micro homogenizer. The extract was centrifuged for 5 min at 15,000 × g. The liquid phase was transferred to a new EP tube and air-dried at 50 °C. Then, dried lipids were dissolved with 200 μl of cholesterol assay buffer. The standard curve was prepared with the cholesterol standard. We added 2 µl of esterase to each well. We added a mixture containing 44 µl of cholesterol assay buffer, 2 µl of substrate mix, and 2 µl of cholesterol enzyme mix in each well-containing samples. The samples were incubated at 37 °C for 30 min. Then, absorbance was measured at 450 nm. If free cholesterol was tested, cholesterol esterase was omitted in the reaction.

### Proteome Profiler^TM^ array

The Human Angiogenesis Array Kit (#ARY007, R&D Systems, Inc., USA) was applied for factor screening. Cell culture supernatant was collected for use. Blocking buffer was added to the membrane for 1 h on a rocking board. The samples were mixed with 15 µl of reconstituted detection antibody cocktail and then incubated at room temperature for 1 h. The mix was transferred onto the membrane for 4 °C overnight. After washing with wash buffer, the cells were incubated with streptavidin-HRP buffer for 30 min. Final detection was performed by adding Chemi Reagent mix and then scanning.

### Enzyme-linked immunosorbent assay

An ELISA kit was used to detect different proteins (PDGF-AA/IGFBP-3 ELISA Kit, # 70-EK1213-96/#70-EK1168-96, MultiSciences; MMP-9/VEGF/ANG ELISA kit, #EK0465/EK0575/, Boster). Cell culture supernatants were collected after treatment. Samples were processed according to the manufacturer’s instructions. In brief, 100 µl samples and standard proteins were added to each well and incubated at 37 °C for 90 min. After the samples were washed, 100 µl of biotin-labeled antibody was added for 60 min at 37 °C. TMB was then applied. Absorbance at 450 nm was measured with a microplate reader (Thermo Fisher Scientific, Waltham, MA, USA)

### Immunoprecipitation assays

Tris-HCl (100 mM, pH 6.8), 20% glycerol, 4% SDS, 1 mM NaF, 1 mM Na3VO4, and 1 mM PMSF were used to prepare cell lysates. The whole procedure was performed according to the manufacturer’s instructions (#10007D, Invitrogen, USA). Cell lysates were incubated with 4 µg of precipitating anti-FASN antibody for 1 h at room temperature. Fifty microliters of A-agarose slurry were added, and then, the mixture was mixed for 1 h at room temperature. The samples were finally resuspended in 20 μL of elution buffer for later immunoblotting.

### Clinical specimens

Informed consent was obtained from each patient. The entire study was approved by the Ethics Committee of the First Affiliated Hospital of Sun Yat-sen University (Guangzhou, China). A total of 142 paraffin-embedded tissues of cervical cancer collected from January 2006 to December 2009 were obtained from the Department of Pathology. All patients were matched from stage Ia2 to IIa2 and underwent radical hysterectomy and lymphadenectomy. None of the patients were treated with chemotherapy or radiotherapy before surgery. Twenty normal uterine cervical tissues as controls were collected from those who underwent hysterectomy for nonmalignant conditions.

### Statistical analysis

Statistical analyses were performed with SPSS 20.0 statistical software (Chicago, IL, USA). All values are shown as the mean ± SD. Statistical differences between mean values were determined by ANOVA, followed by Fisher’s protected least significance difference test. The χ^2^ test and Fisher’s exact test were applied to analyze the relationship between FASN expression and the clinicopathological characteristics. The Kaplan–Meier method and the log-rank test were used in the survival analysis. In all cases, *P* < 0.05 was considered statistically significant.

## Supplementary information

Supplementary Material

Supplemental Material original western blots

Reproducibility checklist

## Data Availability

All data generated or analyzed in this study are included in this paper and can be obtained from the corresponding author according to formal requirement.
