# Supplementary material for: FASN promotes lymph node metastasis in cervical cancer via cholesterol reprogramming and lymphangiogenesis
Source: Cell Death Dis. 2022 May 21;13(5):488. doi: 10.1038/s41419-022-04926-2 (PMC9124199; doi:10.1038/s41419-022-04926-2)
Supplement: Supplementary file 1 — Supplementary Material [file 41419_2022_4926_MOESM1_ESM.pdf]

FASN promotes lymph node metastasis in cervical cancer via cholesterol reprogramming and lymphangiogenesis

Qiqiao Du [1], Pan Liu [1,4], Chunyu Zhang[1], Tianyu Liu [1], Wei Wang [1], Chunliang Shang [2], Jieyu Wu [3], Yuandong Liao [1], Yili Chen [1], Jiaming Huang [1], Hao Tan [1], Yunhe Zhao [1], Meng Xia [1], Junxiu Liu [1]* and Shuzhong Yao [1]*

[1] Department of Obstetrics and Gynecology, The First Affiliated Hospital of Sun Yat-sen University, Guangzhou, P.R. China

[2] Department of Obstetrics and Gynecology, Peking University Third Hospital, Beijing, P.R. China

[3] Department of Microbiology, Tumor and Cell Biology, Karolinska Institute, 171 77 Stockholm, Sweden

[4] Department of Obstetrics and Gynecology, Union Hospital, Tongji Medical College, Huazhong University of Science and Technology, Wuhan, 430022, China.

***Correspondence**: Junxiu Liu liujxiu@mail.sysu.edu.cn; Shuzhong Yao yaoshuzh@mail.sysu.edu.cn

Running title: FASN in cervical cancer nodal metastasis

Supplementary Material

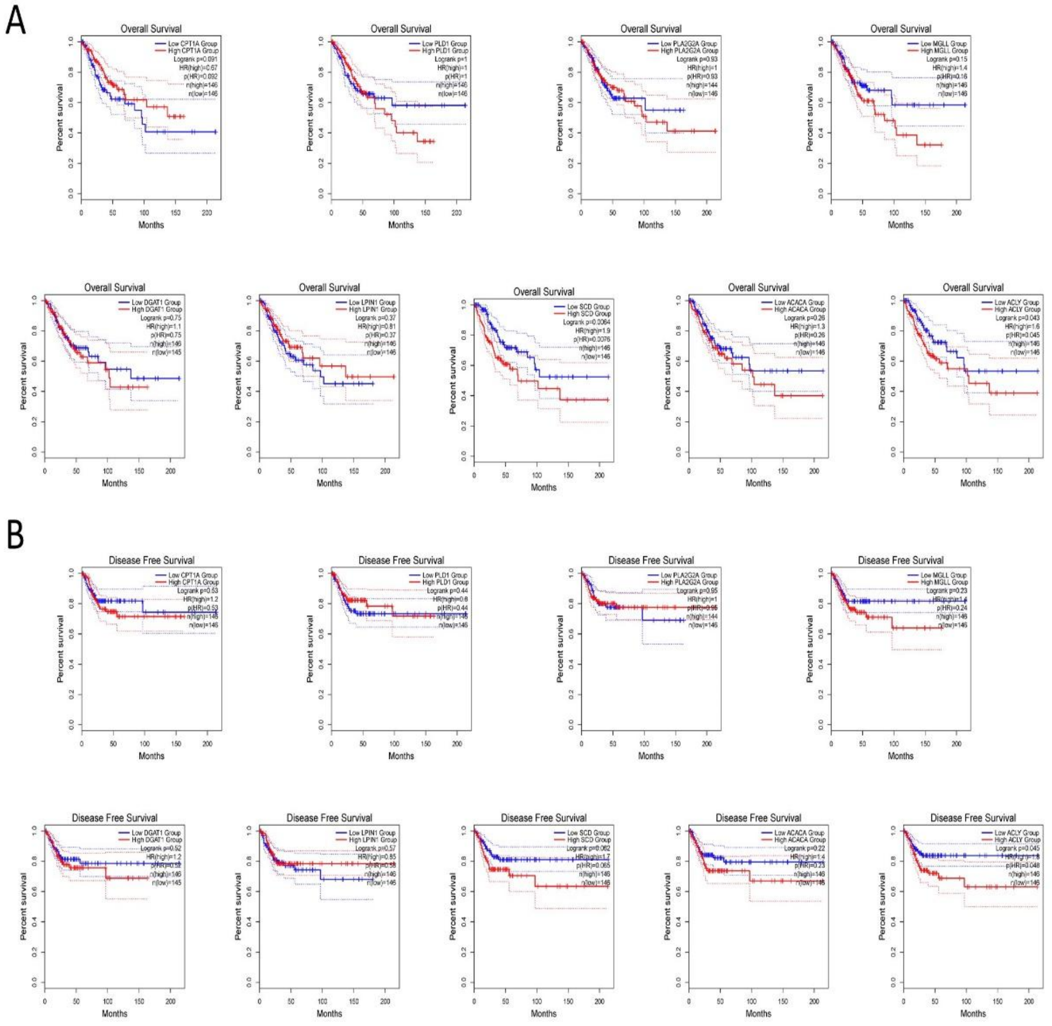

Fig S1. Overall and disease-free survival analysis of lipid metabolism related genes involved in cancer metastasis using TCGA dataset. A. Overall survival analysis of 9 lipid metabolism related genes involved in cancer metastasis using TCGA dataset. **B**. Disease-free survival analysis of 9 lipid metabolism related genes involved in cancer metastasis using TCGA dataset.

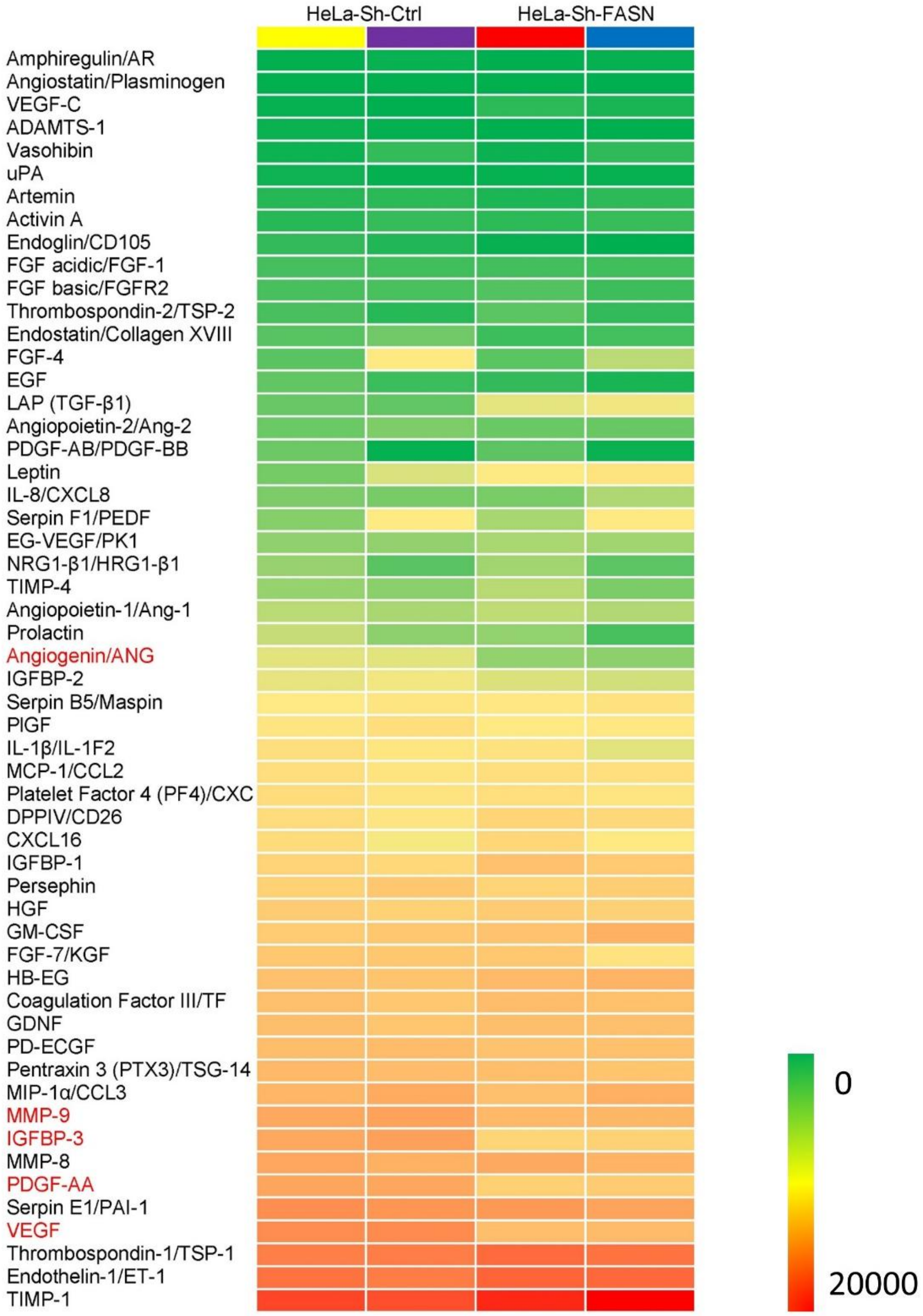

Fig S2. Heatmap of angiogenesis related factors in HeLa cell culture supernatant. The pixels were analyzed and form the heatmap. The red marked the most differentiated ones

Clinicopathologic Variable	Total(N=142)	FASN		P value
		Low expression	High expression	
**Age/years**				
<=42	55	36	19	
>42	87	49	38	0.283
**FIGO stage**				
Ia2	8	4	4	
Ib1	99	63	36	
Ib2	10	5	5	
IIa1	16	6	10	
IIa2	9	7	2	0.527
**Tumor size**				
<=4	114	69	45	
>4	28	16	12	0.746
**Pathologic types**				
Squamous cell cacinoma	113	65	48	
Adenocarcinoma	20	14	6	
Adenosquamous carcinoma	9	6	3	0.276
**Stromal invasion**				
<1/2	55	27	28	
≥1/2	87	58	29	**0.038**
**Lymphovascular space invasion**				
Yes	16	8	8	
No	126	77	49	0.397
**Differentiation grade**				
Well	11	6	5	
Moderate	51	27	24	
Poor	80	52	28	0.175
**Pelvic lymph node metastasis**				
Yes	29	12	17	
No	113	73	40	**0.023**
**Vaginal involvement**				
Yes	3	3	0	
No	139	82	57	0.154
**Parametrial infiltration**				
Yes	2	2	0	
No	140	83	57	0.247
**Recurrence**				
Yes	13	0	13	
No	129	85	44	**<0.001**
**Vital status at follow-up**				
Alive	123	84	39	
Dead	19	1	18	**<0.001**

Abbreviations: FIGO: the International Federation of Gynecology and Obstetrics;

The bold number inside the table reflected P < 0.05

TableS1. Correlation between FASN expression and clinicopathological characteristics of patients with cervical cancer.

Clinicopathologic Variable	Total(N=142)	OS Univariate p value	OS Multivariate RR(95% CI)	OS Multivariate p value	DFS Univariate p value	DFS Multivariate RR(95% CI)	DFS Multivariate p value
**Age/years**							
<=42	55						
>42	87	0.399	N	N	0.393	N	N
**FIGO stage**							
Ia2	8						
Ib1	99						
Ib2	10						
IIa1	16						
IIa2	9	0.630	N	N	0.631	N	N
**Tumor size**							
<=4	114						
>4	28	0.262	N	N	0.268	N	N
**Pathologic types**							
Squamous cell cacinoma	113						
Adenocarcinoma	20						
Adenosquamous carcinoma	9	0.332	N	N	0.336	N	N
**Stromal invasion**							
<1/2	55						
≥1/2	87	0.443	N	N	0.437	N	N
**Differentiation grade**							
Well	11						
Moderate	51						
Poor	80	0.414	N	N	0.409	N	N
**Vaginal involvement**							
Yes	3						
No	139	0.530	N	N	0.529	N	N
**Parametrial infiltration**							
Yes	2						
No	140	0.610	N	N	0.609	N	N
**Lymphovascular space invasion**							
Yes	16						
No	126	**<0.001**	0.639(0.156-2.616)	0.533	**<0.001**	0.764(0.196-2.972)	0.698
**Pelvic-lymph node metastasis**							
Yes	29						
No	113	**<0.001**	0.275(0.092-0817)	**0.020**	**<0.001**	0.275(0.090-0.837)	**0.023**
**Recurrence**							
Yes	13						
No	129	**<0.001**	0.026(0.007-0.095)	**<0.001**	**<0.001**	0.022(0.006-0.081)	**<0.001**
**FASN expression**							
Low	85						
High	57	**<0.001**	0.115(0.013-0.986)	**0.049**	**<0.001**	0.114(0.013-0.983)	**0.048**

Abbreviations: N: Not application; FIGO: the International Federation of Gynecology and Obstetrics; The bold number inside the table reflected P < 0.05

Table S2. Univariate and multivariate analyses of factors associated with overall survival and disease-free survival
