# Supplementary material for: FASN promotes lymph node metastasis in cervical cancer via cholesterol reprogramming and lymphangiogenesis
Source: Cell Death Dis. 2022 May 21;13(5):488. doi: 10.1038/s41419-022-04926-2 (PMC9124199; doi:10.1038/s41419-022-04926-2)
Supplement: Supplementary file 2 — Supplemental Material original western blots [file 41419_2022_4926_MOESM2_ESM.pdf]

Fig 2B FASN

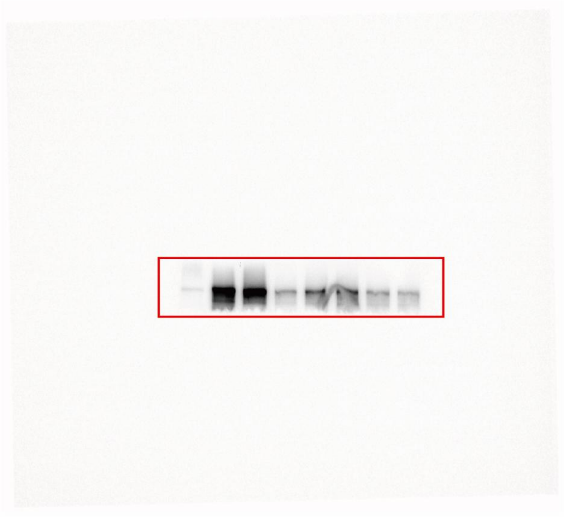

Fig 2B β-actin

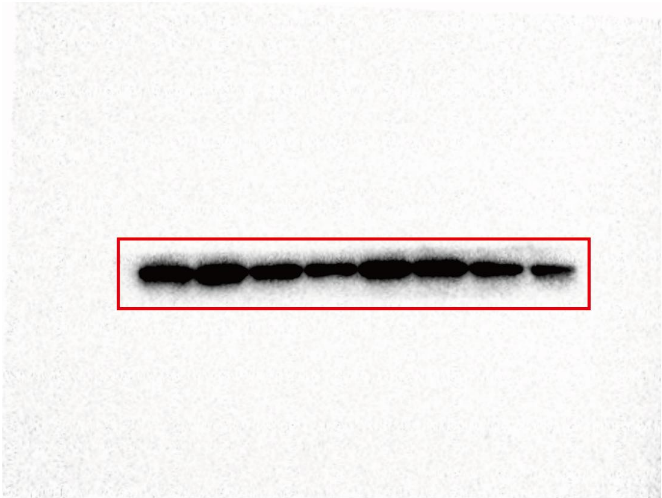

Fig 2D FASN

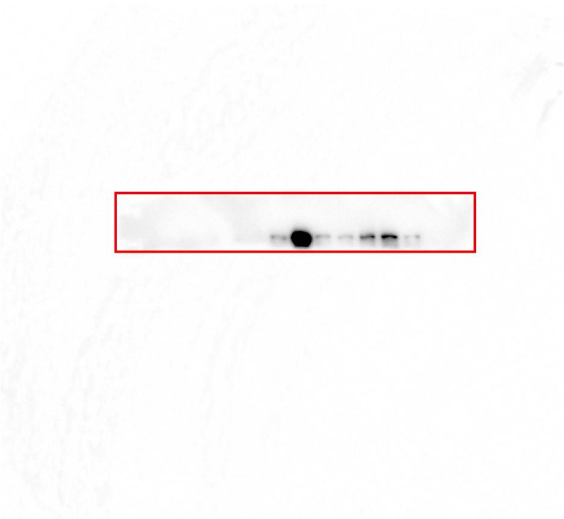

Fig 2D β-actin

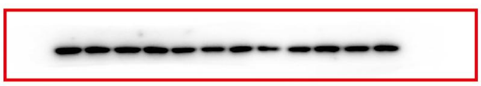

Fig 4J p-Fak HeLa si-FASN#1

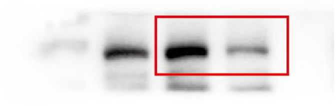

Fig 4J Fak HeLa si-FASN#1

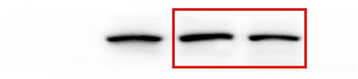

Fig 4J β-actin HeLa si-FASN#1

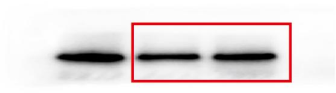

Fig 4J p-Fak HeLa si-FASN#2

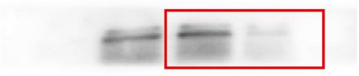

Fig 4J Fak HeLa si-FASN#2

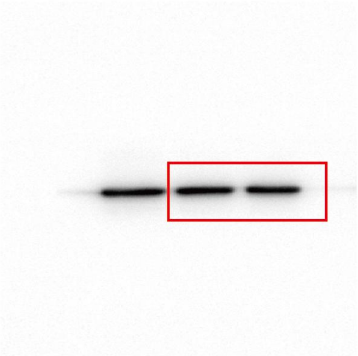

Fig 4J β-actin HeLa si-FASN#2

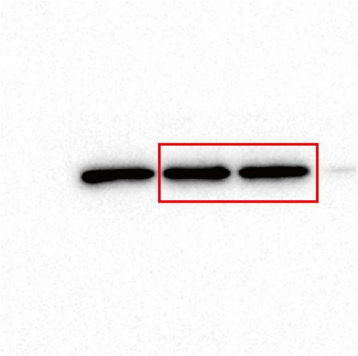

Fig 4J p-Fak CaSki si-FASN#1

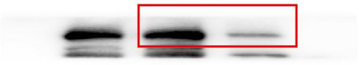

Fig 4J Fak CaSki si-FASN#1

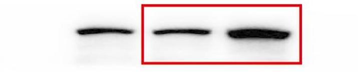

Fig 4J β-actin CaSki si-FASN#1

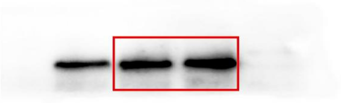

Fig 4J p-Fak CaSki si-FASN#2

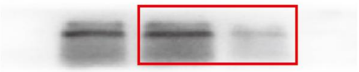

Fig 4J Fak CaSki si-FASN#2

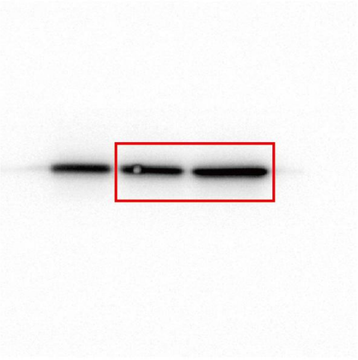

Fig 4J β-actin CaSki si-FASN#2

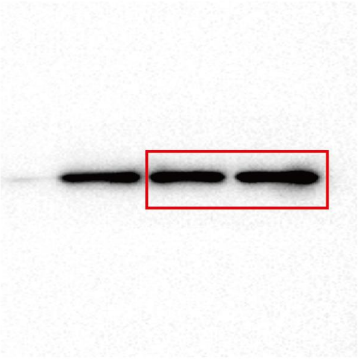

Fig 4K p-Fak C33A

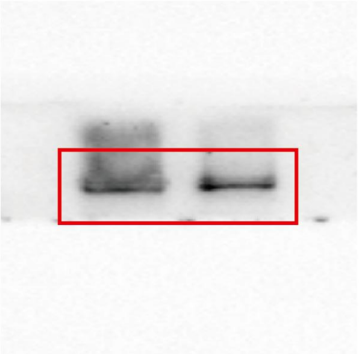

Fig 4K Fak C33A

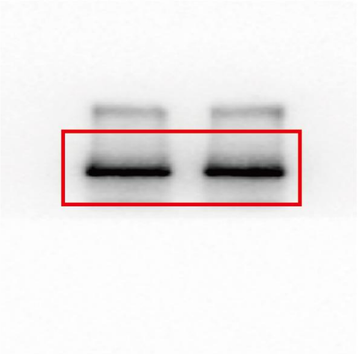

Fig 4K β-actin C33A

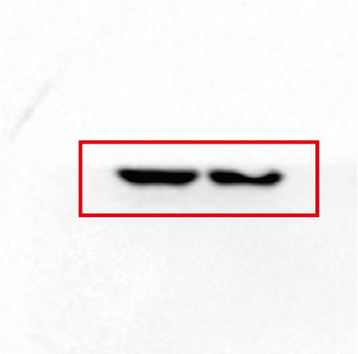

Fig 5A p-Fak

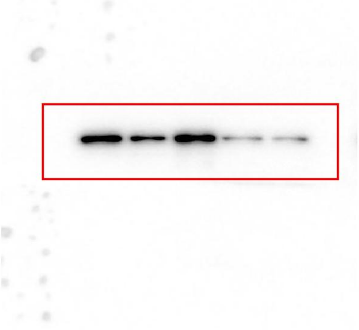

Fig 5A Fak

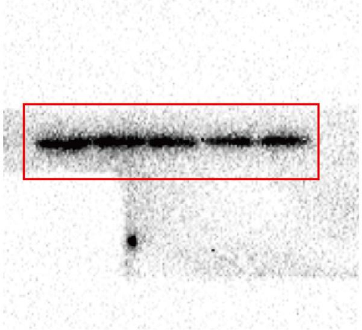

Fig 5A β-actin

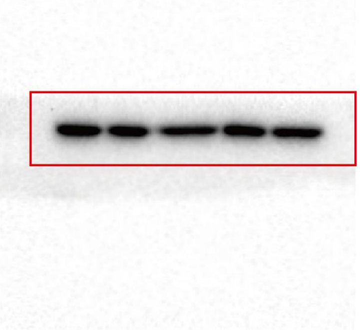

Fig 5B p-Fak

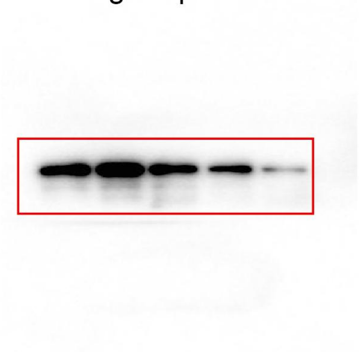

Fig 5B Fak

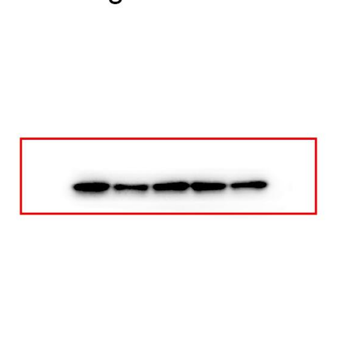

Fig 5B β-actin

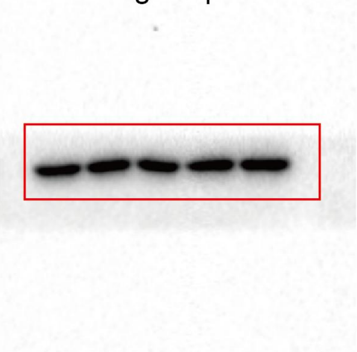

Fig 5C p-c-Src si-FASN#1

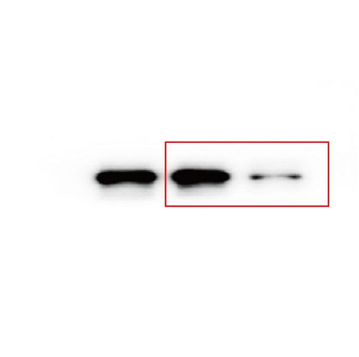

Fig 5C c-Src si-FASN#1

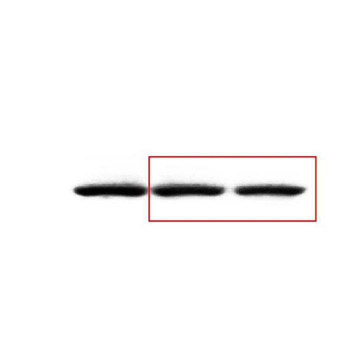

Fig 5C p-Akt si-FASN#1

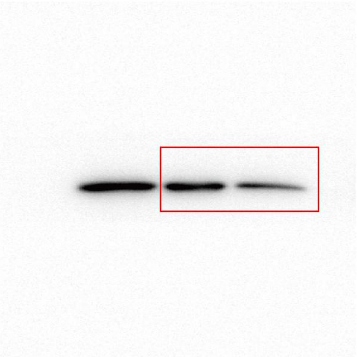

Fig 5C Akt si-FASN#1

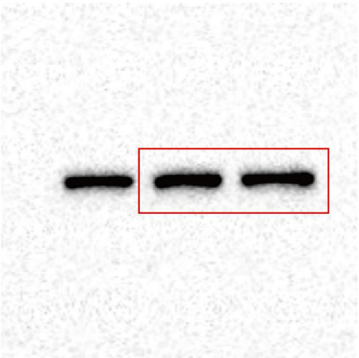

Fig 5C β-actin si-FASN#1

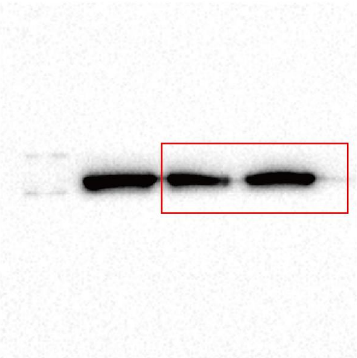

Fig 5C p-c-Src si-FASN#2

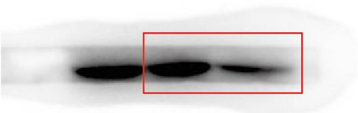

Fig 5C c-Src si-FASN#2

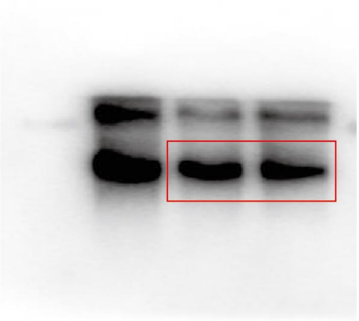

Fig 5C p-Akt si-FASN#2

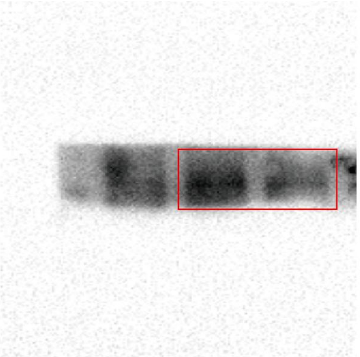

Fig 5C Akt si-FASN#2

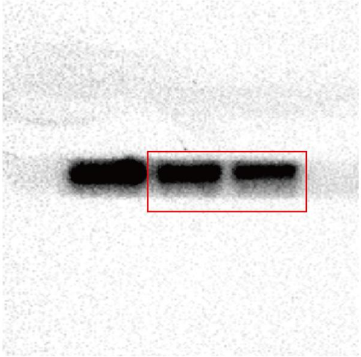

Fig 5C β-actin si-FASN#2

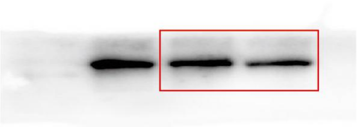

Fig 5D p-c-Src k si-FASN#1

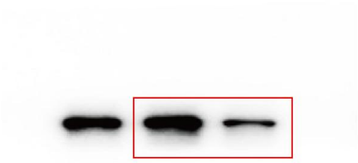

Fig 5D c-Src si-FASN#1

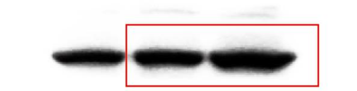

Fig 5D p-Akt si-FASN#1

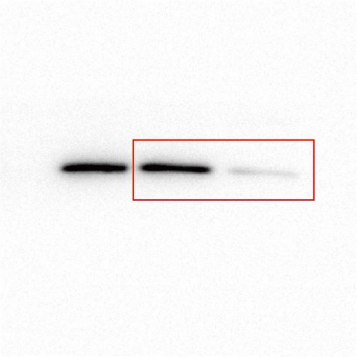

Fig 5D Akt si-FASN#1

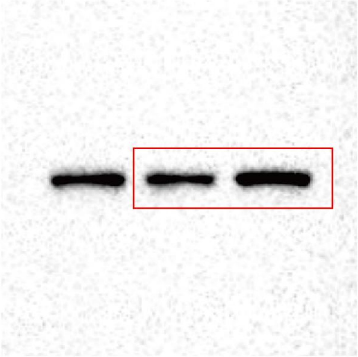

Fig 5D β-actin si-FASN#1

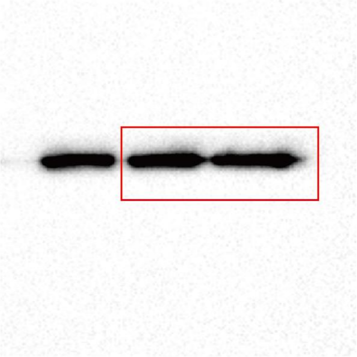

Fig 5D p-c-Src si-FASN#2

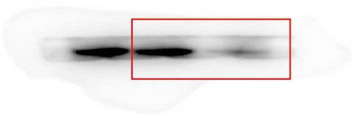

Fig 5D c-Src si-FASN#2

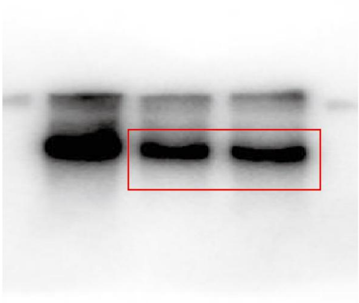

Fig 5D p-Akt si-FASN#2

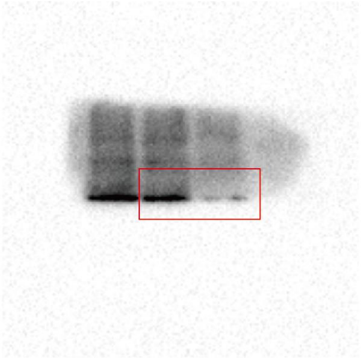

Fig 5D Akt si-FASN#2

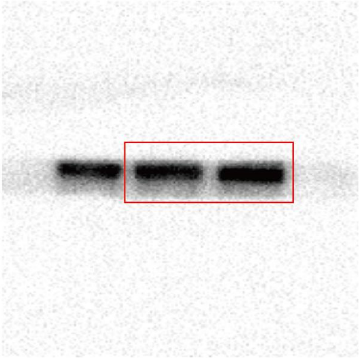

Fig 5D β-actin si-FASN#2

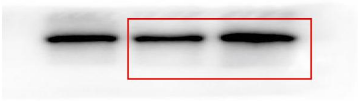

Fig 5E p-c-Src

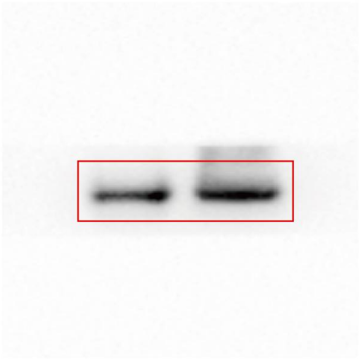

Fig 5E c-Src

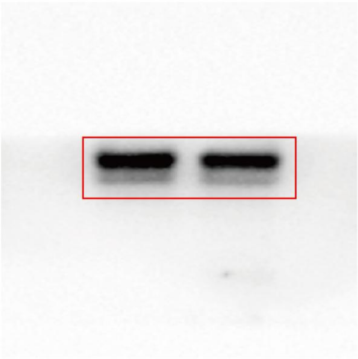

Fig 5E p-Akt

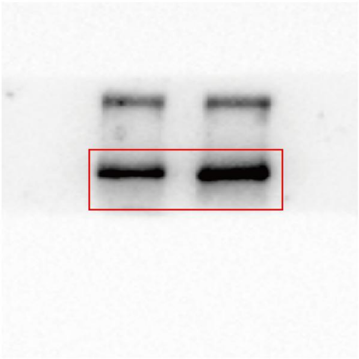

Fig 5E Akt

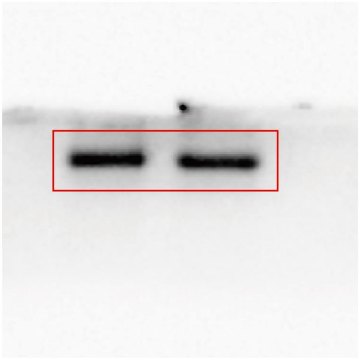

Fig 5E β-actin

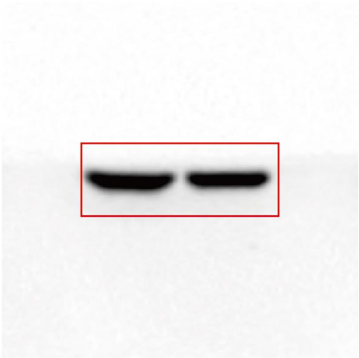

Fig 5F p-Fak

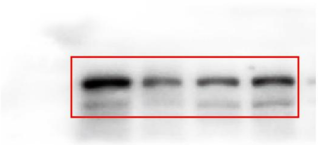

Fig 5F Fak

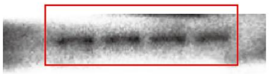

Fig 5F p-Akt

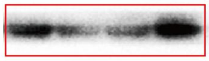

Fig 5F Akt

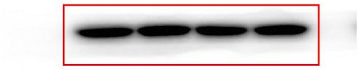

Fig 5F β-actin

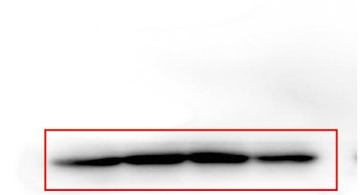

Fig 5G p-Fak

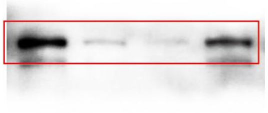

Fig 5G Fak

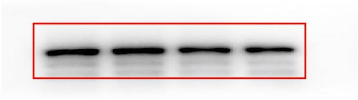

Fig 5G p-Akt

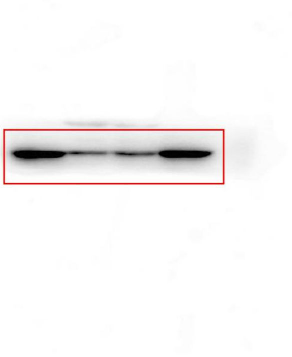

Fig 5G Akt

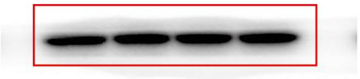

Fig 5G β-actin

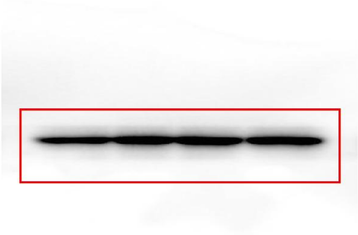

Fig 5H p-Fak

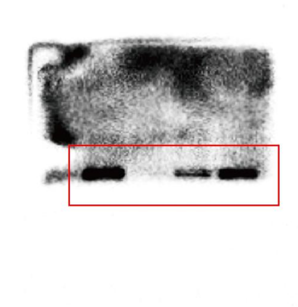

Fig 5H Fak

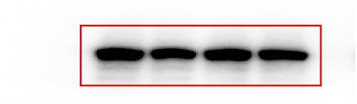

Fig 5H p-c-Src

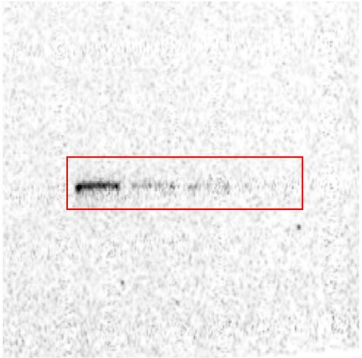

Fig 5H c-Src

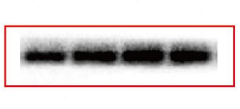

Fig 5H β-actin

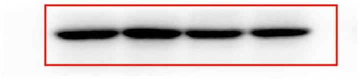

Fig 5I p-Fak

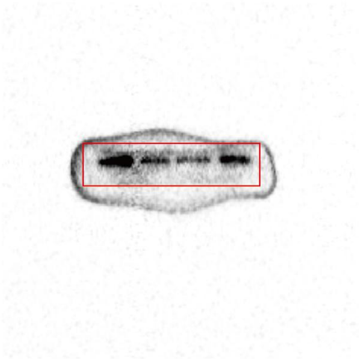

Fig 5I Fak

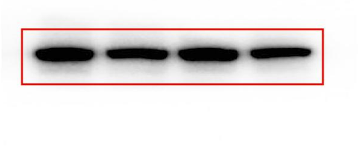

Fig 5I p-c-Src

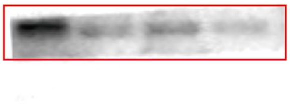

Fig 5I c-Src

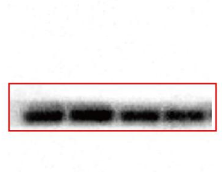

Fig 5I β-actin

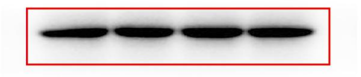

Fig 5J c-Src HeLa

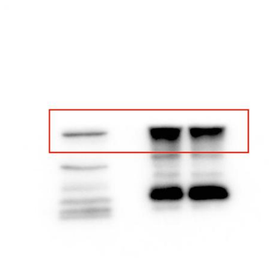

Fig 5J FASN HeLa

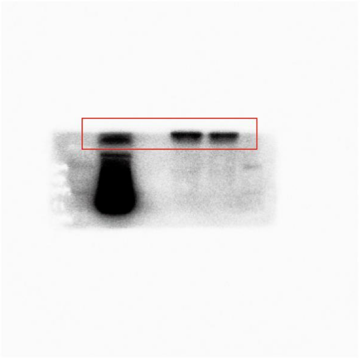

Fig 5J c-Src CaSki

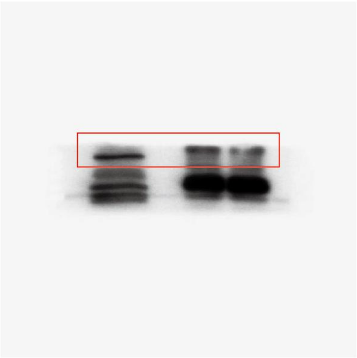

Fig 5J FASN Caski

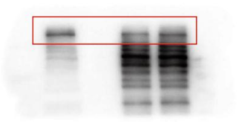

Note: For the western blot gel pictures in Fig 4J, 5C, 5D, the HeLa or CaSki cells were treated with nothing, scrambled-siRNA, FASN-siRNA(Left to right). And only the scrembled-siRNA and FASN-siRNA were cropped to show in the main figures.
